# 5-Bromo-4-(3,4-dimeth­oxy­phen­yl)thia­zol-2-amine

**DOI:** 10.1107/S1600536812019320

**Published:** 2012-05-05

**Authors:** Hazem A. Ghabbour, Tze Shyang Chia, Hoong-Kun Fun

**Affiliations:** a Department of Pharmaceutical Chemistry, College of Pharmacy, King Saud University, PO Box 2457, Riyadh 11451, Saudi Arabia; b X-ray Crystallography Unit, School of Physics, Universiti Sains Malaysia, 11800 USM, Penang, Malaysia

## Abstract

In the title compound, C_11_H_11_BrN_2_O_2_S, the thia­zole ring makes a dihedral angle of 53.16 (11)° with the adjacent benzene ring. The two meth­oxy groups are slightly twisted from the attached benzene ring with C—O—C—C torsion angles of −9.2 (3) and −5.5 (3)°. In the crystal, mol­ecules are linked by a pair of N—H⋯N hydrogen bonds into an inversion dimer with an *R*_2_^2^(8) ring motif. The dimers are further connected by N—H⋯O hydrogen bonds into a tape along [-110].

## Related literature

For applications of the thia­zole ring system, see: Hargrave *et al.* (1983 ▶); Patt *et al.* (1992 ▶); Haviv *et al.* (1988 ▶); Jaen *et al.* (1990 ▶); Tsuji & Ishikawa (1994 ▶); Bell *et al.* (1995 ▶). For applications of amino­thia­zoles, see: Fink *et al.* (1999 ▶); Van Muijlwijk-Koezen *et al.* (2001 ▶); Metzger (1984 ▶). For hydrogen-bond motifs, see: Bernstein *et al.* (1995 ▶). For the preparation, see: Das *et al.* (2006 ▶). For stability of the temperature controller used in the data collection, see: Cosier & Glazer (1986 ▶).
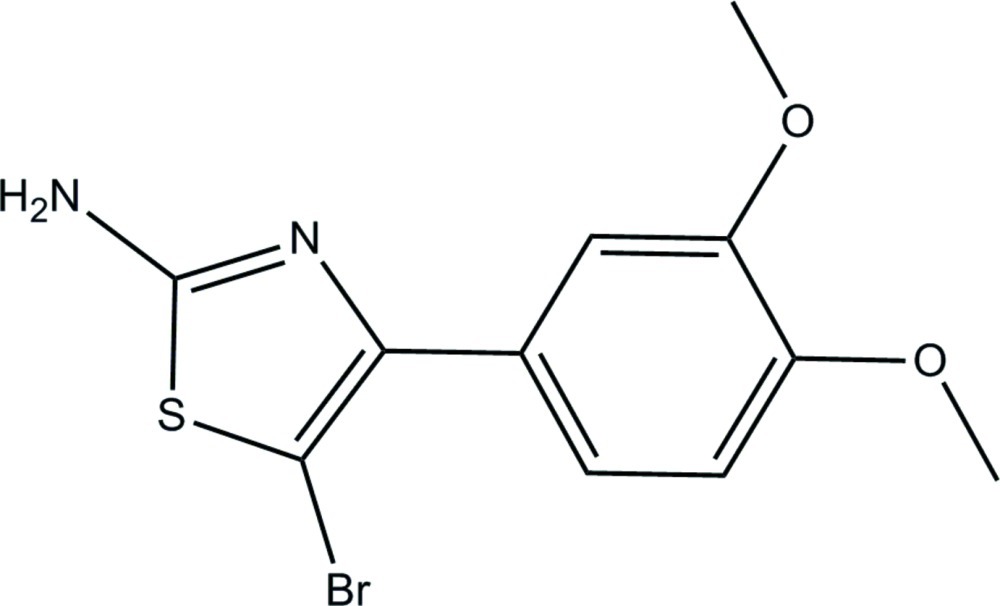


## Experimental

### 

#### Crystal data

C_11_H_11_BrN_2_O_2_S
*M* *_r_* = 315.19
Triclinic, 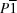

*a* = 7.4873 (2) Å
*b* = 8.0359 (2) Å
*c* = 10.6428 (3) Å
α = 86.571 (2)°
β = 77.633 (2)°
γ = 85.330 (2)°
*V* = 622.82 (3) Å^3^
*Z* = 2
Mo *K*α radiation
μ = 3.46 mm^−1^
*T* = 100 K
0.45 × 0.20 × 0.09 mm

#### Data collection

Bruker SMART APEXII CCD area-detector diffractometer
Absorption correction: multi-scan (*SADABS*; Bruker, 2009 ▶) *T*_min_ = 0.305, *T*_max_ = 0.737
10861 measured reflections
2121 independent reflections
1888 reflections with *I* > 2σ(*I*)
*R*_int_ = 0.030

#### Refinement

*R*[*F* ^2^ > 2σ(*F* ^2^)] = 0.024
*wR*(*F* ^2^) = 0.071
*S* = 1.12
2121 reflections
164 parameters
H atoms treated by a mixture of independent and constrained refinement
Δρ_max_ = 1.17 e Å^−3^
Δρ_min_ = −0.73 e Å^−3^

### 

Data collection: *APEX2* (Bruker, 2009 ▶); cell refinement: *SAINT* (Bruker, 2009 ▶); data reduction: *SAINT*; program(s) used to solve structure: *SHELXTL* (Sheldrick, 2008 ▶); program(s) used to refine structure: *SHELXTL*; molecular graphics: *SHELXTL*; software used to prepare material for publication: *SHELXTL* and *PLATON* (Spek, 2009 ▶).

## Supplementary Material

Crystal structure: contains datablock(s) global, I. DOI: 10.1107/S1600536812019320/is5132sup1.cif

Structure factors: contains datablock(s) I. DOI: 10.1107/S1600536812019320/is5132Isup2.hkl

Supplementary material file. DOI: 10.1107/S1600536812019320/is5132Isup3.cml

Additional supplementary materials:  crystallographic information; 3D view; checkCIF report

## Figures and Tables

**Table 1:** Hydrogen-bond geometry (Å, °)

*D*—H⋯*A*	*D*—H	H⋯*A*	*D*⋯*A*	*D*—H⋯*A*
N2—H2*N*2⋯O1^i^	0.78 (3)	2.40 (3)	2.992 (3)	134 (3)
N2—H2*N*2⋯O2^i^	0.78 (3)	2.37 (3)	3.112 (3)	161 (3)
N2—H1*N*2⋯N1^ii^	0.81 (3)	2.20 (3)	2.998 (3)	168 (3)

Symmetry codes: (i) 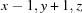; (ii) 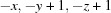.

**Fig. 1.**
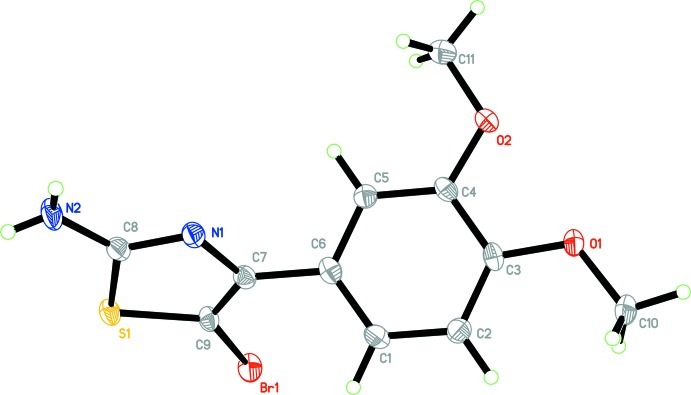
The molecular structure of the title compound with atom labels and 50% probability displacement ellipsoids.

**Fig. 2.**
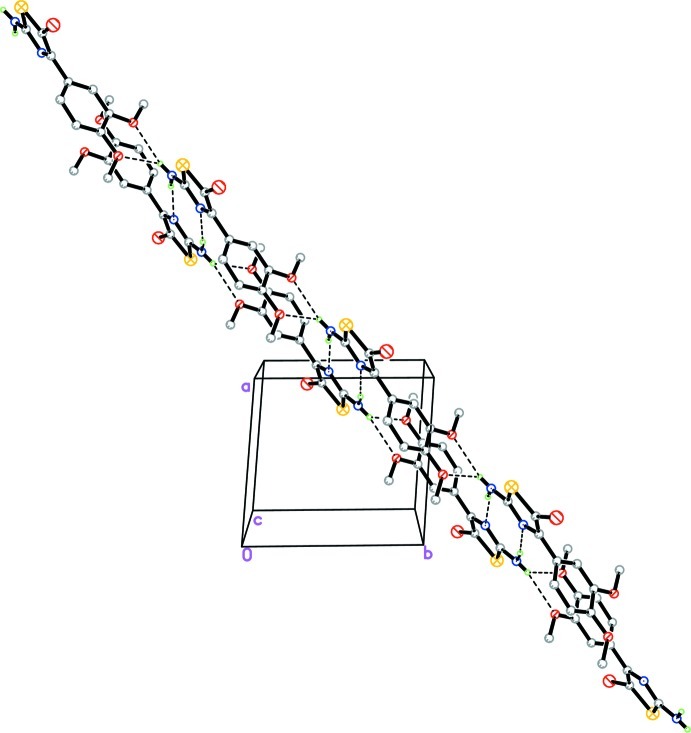
The crystal packing of the title compound. The dashed lines represent the hydrogen bonds. For clarity sake, hydrogen atoms not involved in hydrogen bonding have been omitted.

**Fig. 3.**
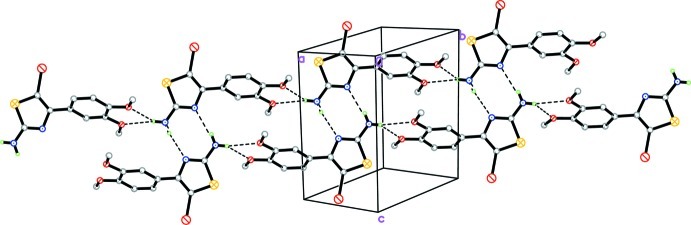
The crystal packing of the title compound viewed along [1 1 0].

## supplementary crystallographic information

### Comment

The thiazole ring system is a useful structural motif found in numerous biologically active molecules. This structure has found applications in drug development for the treatment of allergies (Hargrave *et al.*, 1983), hypertension (Patt *et al.*, 1992), inflammation (Haviv *et al.*, 1988), schizophrenia (Jaen *et al.*, 1990), bacterial (Tsuji & Ishikawa, 1994) and HIV infections (Bell *et al.*, 1995). Aminothiazoles are known to be ligands of estrogen receptors (Fink *et al.*, 1999) as well as a novel class of adenosine receptor antagonists (Van Muijlwijk-Koezen *et al.*, 2001). Other analogues are used as fungicides, inhibiting *in vivo* growth of Xanthomonas, as an ingredient of herbicides or as schistosomicidal and anthelmintic drugs (Metzger, 1984).

In the title compound (Fig. 1), the thiazole ring (S1/N1/C7–C9) makes a dihedral angle of 53.16 (11)° with the adjacent benzene ring (C1–C6). The two methoxy groups (O1/C10 & O2/C11) are slightly twisted from the C1–C6 ring with torsion angles C10—O1—C3—C2 = -9.2 (3) and C11—O2—C4—C5 = -5.5 (3)°.

In the crystal packing (Fig. 2 & 3), the molecules are linked by intermolecular N2—H1N2···N1 hydrogen bonds into dimers with *R*_2_^2^(8) ring motifs (Bernstein *et al.*, 1995). The dimers are further connected by intermolecular N2—H2N2···O1 and N2—H2N2···O2 hydrogen bonds (Table 1) into infinite tapes along [110].

### Experimental

The title compound was prepared from the reaction of 4-(3,4-dimethoxyphenyl)thiazol-2-amine (236 mg, 1 mmol) with bromine (161 mg, 1.1 mmol) in glacial acetic acid and heated at 80 °C for 1.5 h. Single crystals of the title compound suitable for X-ray structure determination were recrystallized from ethanol by the slow evaporation of the solvent at room temperature after several days (Das *et al.*, 2006).

### Refinement

Atom H1N2 and H2N2 were located in a difference Fourier map and refined freely [N—H = 0.80 (3) and 0.78 (3) Å]. The remaining H atoms were positioned geometrically (C—H = 0.95 and 0.98 Å) and refined using a riding model with *U*_iso_(H) = 1.2 or 1.5*U*_eq_(C). A rotating group model was applied to the methyl groups. Three outliers (-2 6 4), (5 -3 8) and (5 -2 9) were omitted.

### Crystal data

**Table:**

C_11_H_11_BrN_2_O_2_S	*Z* = 2
*M**_r_* = 315.19	*F*(000) = 316
Triclinic, *P*1	*D*_x_ = 1.681 Mg m^−^^3^
Hall symbol: -P 1	Mo *K*α radiation, λ = 0.71073 Å
*a* = 7.4873 (2) Å	Cell parameters from 7495 reflections
*b* = 8.0359 (2) Å	θ = 2.6–35.5°
*c* = 10.6428 (3) Å	µ = 3.46 mm^−^^1^
α = 86.571 (2)°	*T* = 100 K
β = 77.633 (2)°	Plate, brown
γ = 85.330 (2)°	0.45 × 0.20 × 0.09 mm
*V* = 622.82 (3) Å^3^	

### Data collection

**Table:**

Bruker SMART APEXII CCD area-detector diffractometer	2121 independent reflections
Radiation source: fine-focus sealed tube	1888 reflections with *I* > 2σ(*I*)
Graphite monochromator	*R*_int_ = 0.030
φ and ω scans	θ_max_ = 25.0°, θ_min_ = 2.0°
Absorption correction: multi-scan (*SADABS*; Bruker, 2009)	*h* = −8→8
*T*_min_ = 0.305, *T*_max_ = 0.737	*k* = −9→9
10861 measured reflections	*l* = −12→12

### Refinement

**Table:**

Refinement on *F*^2^	Primary atom site location: structure-invariant direct methods
Least-squares matrix: full	Secondary atom site location: difference Fourier map
*R*[*F*^2^ > 2σ(*F*^2^)] = 0.024	Hydrogen site location: inferred from neighbouring sites
*wR*(*F*^2^) = 0.071	H atoms treated by a mixture of independent and constrained refinement
*S* = 1.12	*w* = 1/[σ^2^(*F*_o_^2^) + (0.0446*P*)^2^ + 0.1359*P*] where *P* = (*F*_o_^2^ + 2*F*_c_^2^)/3
2121 reflections	(Δ/σ)_max_ = 0.002
164 parameters	Δρ_max_ = 1.17 e Å^−^^3^
0 restraints	Δρ_min_ = −0.73 e Å^−^^3^

### Special details

**Table:**

Experimental. The crystal was placed in the cold stream of an Oxford Cryosystems Cobra open-flow nitrogen cryostat (Cosier & Glazer, 1986) operating at 100.0 (1) K.
Geometry. All e.s.d.'s (except the e.s.d. in the dihedral angle between two l.s. planes) are estimated using the full covariance matrix. The cell e.s.d.'s are taken into account individually in the estimation of e.s.d.'s in distances, angles and torsion angles; correlations between e.s.d.'s in cell parameters are only used when they are defined by crystal symmetry. An approximate (isotropic) treatment of cell e.s.d.'s is used for estimating e.s.d.'s involving l.s. planes.
Refinement. Refinement of *F*^2^ against ALL reflections. The weighted *R*-factor *wR* and goodness of fit *S* are based on *F*^2^, conventional *R*-factors *R* are based on *F*, with *F* set to zero for negative *F*^2^. The threshold expression of *F*^2^ > σ(*F*^2^) is used only for calculating *R*-factors(gt) *etc*. and is not relevant to the choice of reflections for refinement. *R*-factors based on *F*^2^ are statistically about twice as large as those based on *F*, and *R*- factors based on ALL data will be even larger.

### Fractional atomic coordinates and isotropic or equivalent isotropic displacement parameters (Å^2^)

**Table:**

	*x*	*y*	*z*	*U*_iso_*/*U*_eq_	
Br1	−0.16418 (3)	0.27703 (3)	1.04837 (2)	0.02360 (12)	
S1	−0.30599 (8)	0.51061 (8)	0.84026 (6)	0.01865 (17)	
O1	0.6319 (2)	−0.1031 (2)	0.74403 (16)	0.0203 (4)	
O2	0.4088 (2)	−0.1521 (2)	0.60126 (16)	0.0213 (4)	
N1	−0.0368 (3)	0.4074 (3)	0.6600 (2)	0.0187 (5)	
N2	−0.2487 (4)	0.5970 (3)	0.5852 (2)	0.0237 (5)	
C1	0.2811 (3)	0.2379 (3)	0.8495 (2)	0.0195 (6)	
H1A	0.2539	0.3246	0.9090	0.023*	
C2	0.4415 (3)	0.1345 (3)	0.8433 (2)	0.0193 (6)	
H2A	0.5241	0.1524	0.8968	0.023*	
C3	0.4794 (3)	0.0060 (3)	0.7588 (2)	0.0168 (5)	
C4	0.3576 (3)	−0.0193 (3)	0.6795 (2)	0.0162 (5)	
C5	0.2018 (3)	0.0867 (3)	0.6835 (2)	0.0166 (5)	
H5A	0.1218	0.0719	0.6274	0.020*	
C6	0.1613 (3)	0.2164 (3)	0.7706 (2)	0.0169 (5)	
C7	−0.0059 (3)	0.3294 (3)	0.7743 (2)	0.0167 (5)	
C8	−0.1867 (3)	0.5072 (3)	0.6792 (2)	0.0180 (6)	
C9	−0.1366 (3)	0.3686 (3)	0.8796 (2)	0.0174 (5)	
C10	0.7724 (3)	−0.0657 (3)	0.8075 (2)	0.0233 (6)	
H10A	0.8799	−0.1439	0.7821	0.035*	
H10B	0.7274	−0.0762	0.9010	0.035*	
H10C	0.8065	0.0488	0.7831	0.035*	
C11	0.2828 (4)	−0.1928 (4)	0.5250 (3)	0.0260 (6)	
H11A	0.3359	−0.2877	0.4723	0.039*	
H11B	0.2589	−0.0961	0.4689	0.039*	
H11C	0.1675	−0.2225	0.5819	0.039*	
H2N2	−0.324 (4)	0.669 (4)	0.602 (3)	0.028 (9)*	
H1N2	−0.182 (4)	0.606 (4)	0.515 (3)	0.033 (9)*	

### Atomic displacement parameters (Å^2^)

**Table:**

	*U*^11^	*U*^22^	*U*^33^	*U*^12^	*U*^13^	*U*^23^
Br1	0.02354 (18)	0.02661 (19)	0.01783 (16)	0.00291 (11)	−0.00083 (11)	0.00253 (12)
S1	0.0173 (3)	0.0182 (4)	0.0182 (3)	0.0037 (3)	−0.0003 (3)	−0.0012 (3)
O1	0.0161 (9)	0.0188 (10)	0.0266 (9)	0.0041 (7)	−0.0071 (7)	−0.0039 (8)
O2	0.0207 (9)	0.0200 (10)	0.0238 (9)	0.0045 (8)	−0.0060 (8)	−0.0086 (8)
N1	0.0190 (11)	0.0185 (12)	0.0178 (10)	0.0028 (9)	−0.0033 (9)	−0.0020 (9)
N2	0.0244 (14)	0.0241 (15)	0.0187 (12)	0.0125 (11)	−0.0005 (10)	−0.0019 (11)
C1	0.0203 (14)	0.0167 (14)	0.0208 (12)	−0.0003 (11)	−0.0019 (10)	−0.0048 (11)
C2	0.0187 (13)	0.0199 (15)	0.0202 (13)	−0.0014 (11)	−0.0057 (10)	−0.0008 (11)
C3	0.0142 (13)	0.0132 (13)	0.0209 (12)	0.0013 (10)	−0.0003 (10)	0.0021 (10)
C4	0.0178 (13)	0.0135 (14)	0.0152 (11)	−0.0001 (10)	0.0006 (10)	−0.0003 (10)
C5	0.0172 (13)	0.0173 (14)	0.0149 (11)	−0.0009 (10)	−0.0029 (10)	0.0007 (10)
C6	0.0188 (13)	0.0137 (13)	0.0162 (11)	−0.0003 (10)	−0.0001 (10)	0.0030 (10)
C7	0.0175 (13)	0.0136 (13)	0.0190 (12)	−0.0011 (10)	−0.0039 (10)	−0.0008 (11)
C8	0.0193 (14)	0.0170 (14)	0.0171 (12)	0.0013 (11)	−0.0030 (10)	−0.0024 (11)
C9	0.0192 (13)	0.0136 (14)	0.0183 (12)	0.0021 (10)	−0.0035 (10)	0.0017 (11)
C10	0.0140 (13)	0.0262 (16)	0.0296 (14)	0.0026 (11)	−0.0050 (11)	−0.0034 (12)
C11	0.0263 (15)	0.0265 (16)	0.0270 (14)	0.0004 (12)	−0.0080 (12)	−0.0097 (12)

### Geometric parameters (Å, º)

**Table:**

Br1—C9	1.876 (2)	C2—C3	1.382 (3)
S1—C9	1.738 (3)	C2—H2A	0.9500
S1—C8	1.755 (2)	C3—C4	1.402 (3)
O1—C3	1.369 (3)	C4—C5	1.382 (4)
O1—C10	1.426 (3)	C5—C6	1.407 (3)
O2—C4	1.372 (3)	C5—H5A	0.9500
O2—C11	1.437 (3)	C6—C7	1.480 (4)
N1—C8	1.312 (3)	C7—C9	1.355 (3)
N1—C7	1.390 (3)	C10—H10A	0.9800
N2—C8	1.340 (4)	C10—H10B	0.9800
N2—H2N2	0.78 (3)	C10—H10C	0.9800
N2—H1N2	0.80 (3)	C11—H11A	0.9800
C1—C6	1.380 (3)	C11—H11B	0.9800
C1—C2	1.395 (4)	C11—H11C	0.9800
C1—H1A	0.9500		
			
C9—S1—C8	88.28 (12)	C1—C6—C7	121.1 (2)
C3—O1—C10	116.65 (19)	C5—C6—C7	119.8 (2)
C4—O2—C11	117.3 (2)	C9—C7—N1	114.3 (2)
C8—N1—C7	111.7 (2)	C9—C7—C6	126.9 (2)
C8—N2—H2N2	120 (2)	N1—C7—C6	118.7 (2)
C8—N2—H1N2	119 (2)	N1—C8—N2	123.9 (2)
H2N2—N2—H1N2	116 (3)	N1—C8—S1	114.24 (19)
C6—C1—C2	121.1 (2)	N2—C8—S1	121.9 (2)
C6—C1—H1A	119.4	C7—C9—S1	111.44 (19)
C2—C1—H1A	119.4	C7—C9—Br1	128.9 (2)
C3—C2—C1	119.6 (2)	S1—C9—Br1	119.41 (13)
C3—C2—H2A	120.2	O1—C10—H10A	109.5
C1—C2—H2A	120.2	O1—C10—H10B	109.5
O1—C3—C2	124.9 (2)	H10A—C10—H10B	109.5
O1—C3—C4	115.1 (2)	O1—C10—H10C	109.5
C2—C3—C4	120.0 (2)	H10A—C10—H10C	109.5
O2—C4—C5	125.4 (2)	H10B—C10—H10C	109.5
O2—C4—C3	114.6 (2)	O2—C11—H11A	109.5
C5—C4—C3	120.1 (2)	O2—C11—H11B	109.5
C4—C5—C6	120.1 (2)	H11A—C11—H11B	109.5
C4—C5—H5A	119.9	O2—C11—H11C	109.5
C6—C5—H5A	119.9	H11A—C11—H11C	109.5
C1—C6—C5	119.1 (2)	H11B—C11—H11C	109.5
			
C6—C1—C2—C3	−1.4 (4)	C8—N1—C7—C9	1.5 (3)
C10—O1—C3—C2	−9.2 (3)	C8—N1—C7—C6	−178.0 (2)
C10—O1—C3—C4	170.4 (2)	C1—C6—C7—C9	−52.8 (4)
C1—C2—C3—O1	179.9 (2)	C5—C6—C7—C9	128.5 (3)
C1—C2—C3—C4	0.3 (4)	C1—C6—C7—N1	126.6 (2)
C11—O2—C4—C5	−5.5 (3)	C5—C6—C7—N1	−52.1 (3)
C11—O2—C4—C3	175.2 (2)	C7—N1—C8—N2	−179.6 (2)
O1—C3—C4—O2	1.3 (3)	C7—N1—C8—S1	−1.3 (3)
C2—C3—C4—O2	−179.1 (2)	C9—S1—C8—N1	0.6 (2)
O1—C3—C4—C5	−178.0 (2)	C9—S1—C8—N2	179.0 (2)
C2—C3—C4—C5	1.6 (4)	N1—C7—C9—S1	−1.0 (3)
O2—C4—C5—C6	178.4 (2)	C6—C7—C9—S1	178.36 (19)
C3—C4—C5—C6	−2.4 (3)	N1—C7—C9—Br1	172.86 (17)
C2—C1—C6—C5	0.6 (4)	C6—C7—C9—Br1	−7.7 (4)
C2—C1—C6—C7	−178.1 (2)	C8—S1—C9—C7	0.28 (19)
C4—C5—C6—C1	1.3 (3)	C8—S1—C9—Br1	−174.28 (14)
C4—C5—C6—C7	−180.0 (2)		

### Hydrogen-bond geometry (Å, º)

**Table:**

*D*—H···*A*	*D*—H	H···*A*	*D*···*A*	*D*—H···*A*
N2—H2*N*2···O1^i^	0.78 (3)	2.40 (3)	2.992 (3)	134 (3)
N2—H2*N*2···O2^i^	0.78 (3)	2.37 (3)	3.112 (3)	161 (3)
N2—H1*N*2···N1^ii^	0.81 (3)	2.20 (3)	2.998 (3)	168 (3)

Symmetry codes: (i) *x*−1, *y*+1, *z*; (ii) −*x*, −*y*+1, −*z*+1.
